# Effects of Metal Doping on the Structural, Electrical, and Optical Properties of Spin-Coated Nanocrystalline ZnO

**DOI:** 10.3390/nano16171097

**Published:** 2026-09-01

**Authors:** Yusof-den Jamasali, Abdul Mannan Majeed, Algirdas Mekys, Vidas Pakštas, Saulius Miasojedovas, Gediminas Kreiza, Patrik Ščajev

**Affiliations:** 1 Institute of Photonics and Nanotechnology, Faculty of Physics, Vilnius University, Saulėtekio Ave. 3, LT-10257 Vilnius, Lithuania; mannan.majeed@ff.vu.lt (A.M.M.); algirdas.mekys@ff.vu.lt (A.M.); saulius.miasojedovas@ff.vu.lt (S.M.); gediminas.kreiza@ff.vu.lt (G.K.); 2 Department of Physics, College of Natural Sciences and Mathematics, Mindanao State University, Marawi City 9200, Lanao del Sur, Philippines; 3 Department of Physics, Faculty of Science, Kastamonu University, 37200 Kastamonu, Turkey; 4 Department of Characterization of Materials Structure, Center for Physical Sciences and Technology, Saulėtekio Ave. 3, LT-10257 Vilnius, Lithuania; vidas.pakstas@ftmc.lt

**Keywords:** ZnO, elemental doping, spin-coating, structural, optical and electronic properties

## Abstract

In this work, we systematically investigate the effects of metal doping on the structural, electrical, and optical properties of spin-coated nanocrystalline ZnO thin films prepared by a simple acetate-based solution process. The incorporation of different metal dopants significantly modified the crystallographic, electrical, and photoluminescence properties of ZnO. Doping with alkali metals enhanced the photoluminescence efficiency and enabled amplified spontaneous emission, whereas Mg was the only dopant that produced a pronounced blue shift in the photoluminescence spectra. Lithium-doped ZnO exhibited a strong concentration-dependent electrical behavior, producing highly conductive *n*-type ZnO at a 1% doping level and *p*-type conductivity at an 8% concentration. Strong *n*-type conductivity was also achieved using low concentrations of Li and Na and higher concentrations of Al. In contrast, Fe-, Ni-, Cu-, and Pb-doped ZnO exhibited a substantial reduction in electrical conductivity accompanied by strong photoluminescence quenching, indicating enhanced defect-related carrier compensation. These results demonstrate that metal doping provides an effective approach for tailoring the structural, optical, and electrical properties of ZnO and offers a versatile route toward engineering ZnO-based layers for optoelectronic devices, transparent conductive contacts, photodetectors, solar cells, and ultraviolet laser applications.

## 1. Introduction

Metal oxide semiconductors have attracted considerable attention owing to their unique combination of optical, electrical, and chemical properties. Among them, zinc oxide (ZnO) is one of the most extensively studied materials [1,2,3,4,5,6,7,8] because of its wide direct bandgap (3.37 eV), large exciton binding energy (~60 meV, comparable to that of diamond, 80 meV [9]), high optical transparency, low cost, and environmental friendliness [10]. These characteristics make ZnO a promising material for numerous applications, including ultraviolet photodetectors [11,12,13,14], light-emitting diodes [2,14], lasers [15], gas and biosensors [16,17,18,19,20,21,22,23], photocatalysis [24,25,26,27], transparent conductive electrodes [28], piezoelectric energy harvesters [29], and photovoltaic devices [30]. More recently, nanocrystalline ZnO has also been proposed as a seed and transparent contact layer for low-temperature growth of GeSn infrared lasers because it promotes improved crystallization of GeSn alloys [31].

One of the major advantages of ZnO is that its structure, optical absorption, bandgap, and electrical properties can be tailored through elemental substitution or doping [32,33]. Appropriate dopants modify the carrier concentration, crystal structure, defect density, optical bandgap, and radiative recombination efficiency, thereby enabling optimization of ZnO for specific applications. In particular, donor doping using group III elements such as Al, Ga, and In provides highly conductive *n*-type ZnO, making these materials attractive as transparent conductive oxides and potential alternatives to indium tin oxide (ITO) [27]. Conversely, achieving stable and reproducible *p*-type ZnO remains one of the major challenges limiting the development of ZnO-based optoelectronic devices. Group I elements, including Li, Na, and K, together with Cu, Ag, and several group V elements, have therefore been extensively investigated as potential acceptor dopants [34,35,36,37].

Numerous studies have demonstrated that different dopants substantially influence ZnO properties. Magnesium generally increases the bandgap energy and shifts the optical emission toward shorter wavelengths [38], whereas transition metals such as Fe and Cu usually produce red shifts and introduce defect-related recombination centers [39]. Li and Na have been reported to improve the electrical conductivity and optical transparency of ZnO [35,40], while Al is one of the most efficient donor dopants for obtaining highly conductive *n*-type material [41,42]. Similarly, Pb, Ni, Ca, and other elements have been shown to modify the crystal structure, optical absorption, luminescence, and electrical conductivity of ZnO, although the reported effects often depend strongly on the synthesis technique and dopant concentrations [43,44,45].

ZnO thin films have been synthesized using a wide variety of techniques, including chemical vapor deposition, hydrothermal synthesis, combustion synthesis, spray pyrolysis, precipitation, laser ablation, solvothermal growth, and spin coating [40,45,46,47,48,49,50,51,52,53,54,55,56,57,58,59,60,61,62]. Among these methods, spin coating is particularly attractive because it is inexpensive, scalable, compatible with large-area substrates, and enables precise control of composition through solution chemistry [63,64].

Although numerous reports have investigated individual dopants [64,65,66,67,68,69,70], direct comparison of multiple metal alloys prepared under identical synthesis conditions remains limited to a few selected metals [71,72,73]. Consequently, it is often difficult to distinguish intrinsic effects of different dopants from variations introduced by the fabrication method. A systematic comparison performed using the same synthesis route, identical processing conditions, and consistent characterization techniques is therefore needed to establish the relative influence of different metal doping on ZnO properties.

In this work, we systematically investigate the effects of replacing Zn with representative alkali metals (Li, Na, and K), an alkaline-earth metal (Mg), transition metals (Fe, Ni, and Cu), and post-transition metals (Al and Pb) in spin-coated nanocrystalline ZnO thin films prepared by a simple acetate-based solution process. The structural, optical, and electrical properties of the films were characterized by X-ray diffraction (XRD), scanning electron microscopy (SEM), optical transmission spectroscopy, photoluminescence spectroscopy, and electrical measurements. The results provide a side-by-side comparison of nine dopants at four nominal concentrations under a common deposition protocol, together with correlated structural, optical, and electrical measurements. Identical processing reveals systematic dopant-dependent correlations between crystallinity, photoluminescence response, carrier type, and conductivity that cannot be extracted reliably from the separate literature reports [65,66,67,68,69].

## 2. Materials and Methods

### 2.1. Preparation of Samples

ZnO precursor solutions containing Li, Na, K, Mg, Fe, Ni, Cu, Pb, or Al dopants were prepared with nominal dopant concentrations of 1, 2, 4, and 8 at%. The precursor solutions were synthesized using an acetate-based route by partially replacing zinc acetate with the corresponding metal acetate precursor while maintaining the total metal concentration constant.

Zinc acetate dihydrate (Zn(CH_3_COO)_2_ · 2H_2_O, ≥99%), nickel acetate tetrahydrate (Ni(CH_3_COO)_2_ · 3H_2_O, ≥99%), magnesium acetate tetrahydrate (Mg(CH_3_COO)_2_ · 3H_2_O, ≥99%), lead acetate tetrahydrate (Pb(CH_3_COO)_2_ · 3H_2_O, ≥99%), lithium hydroxide monohydrate (Li(OH). H_2_O, ≥99%) were purchased from Sigma-Aldrich (St. Louis, MO, USA). The acetylacetone reagent was purchased from VWR Chemicals (Radnor, PA, USA). Acetone (99%), hydrogen peroxide (34–36%), potassium hydroxide solution (1 M), acetic acid (35%), hydrochloric acid (concentrated), and sodium hydroxide (1 M) were purchased from Honeywell International Inc. (Charlotte, NC, USA).

A 0.1 M ZnO solution was prepared from zinc acetate dihydrate (Zn(CH_3_COO)_2_ · 2H_2_O) in 1 mL ethanol and 10 µL of acetylacetone. The prepared solution was heated at 50 °C for 1 h. For Ni, Mg, and Pb dopants, Ni(CH_3_COO)_2_ · 3H_2_O, Mg(CH_3_COO)_2_ · 3H_2_O and Pb(CH_3_COO)_2_ · 3H_2_O we added with 1%, 2%, 4% and 8% mol into Zn(CH_3_COO)_2_ · 2H_2_O powder and prepared the solutions. The Na-, Li-, Fe-, Cu-, K-, and Al-doped solutions were prepared as described in previous studies [74,75,76,77,78]. In short, all metal dopants were reacted with acetic acid, converted into metal-assisted solutions, then heated at different temperatures in open air to evaporate the solvent, yielding dried metal acetate powders. H_2_O_2_ was also added to the solutions to make the reaction faster. These prepared salts were then added to Zn(CH_3_COO)_2_ · 2H_2_O with different molar percentages (1%, 2%, 4%, and 8%) to get doped precursor solutions.

Commercial glass substrates from Avantor™ (Radnor, PA, USA) were cut into 15 × 20 mm^2^ pieces. Before deposition, the substrates were cleaned ultrasonically for 15 min in a soap solution, followed by three washes: distilled water, acetone, and isopropanol. The cleaned substrates were dried with nitrogen gas and subsequently treated by UV–ozone exposure to improve surface wettability.

Doped ZnO thin films were deposited by spin coating [78]. A 35 μL aliquot of the precursor solution was dispensed onto each substrate and spun at 3000 rpm for 20 s. The deposited layer was dried on a hot plate at 250 °C for 10 min. This deposition–drying cycle was repeated five times to obtain the desired film thickness. Finally, the samples were annealed at 500 °C for 2 h in air. For electrical characterization, silver contacts were deposited by thermal evaporation and connected to external wires using conductive silver paste.

### 2.2. Characterization Techniques

The X-ray diffraction (XRD) patterns of the undoped and doped ZnO films were collected using a SmartLab diffractometer (Rigaku, Tokyo, Japan) equipped with a 9 kW Cu rotating anode X-ray source and an SC-70 scintillation detector (Rigaku, Tokyo, Japan). Grazing-incidence X-ray diffraction (GIXRD) measurements were performed at a fixed incidence angle (ω) of 0.5°, defined as the angle between the incident X-ray beam and the sample surface. Phase identification was carried out using PDXL software version 3.0 (Rigaku, Tokyo, Japan) together with the ICDD PDF-4+ database (2025 release). The crystallite size of the undoped and doped ZnO films was estimated from diffraction peak broadening using the Halder–Wagner method implemented in the PDXL software package [79]. The surface morphology and cross-sectional structure of the films were examined using a Hitachi SU8230 (Hitachi High-Tech Corporation, Tokyo, Japan) scanning electron microscope (SEM). Optical transmission spectra were recorded using an Edinburgh Instruments FLS980 spectrometer (Livingston, UK).

Photoluminescence (PL) measurements were performed using a time-resolved photoluminescence system consisting of a Hamamatsu C10627 streak camera (Hamamatsu, Japan) coupled to a 30 cm Acton monochromator (SP2300, Princeton Instruments, (Acton, MA, USA)) in a backscattering configuration. The samples were excited with 325 nm laser pulses generated by an Orpheus optical parametric amplifier (Light Conversion, Vilnius, Lithuania) and pumped by a PHAROS femtosecond laser (Light Conversion, Vilnius, Lithuania). The PL spectra were extracted by integrating the emission over a 200 ps time window in order to suppress the contribution from the glass substrate. The excitation fluence was varied using continuously variable neutral-density filters.

The electrical properties of the films were investigated using current–voltage (I–V) measurements and conductivity determination based on the Seebeck effect under an applied thermal gradient [80]. Electrical conductivity was determined from the measured I–V characteristics. Resistivity and volt-amperic (IV) measurements were performed using the 4-point probe technique with Keithley 6430 and Keithley 6514 multimeters (TEKTRONIX, INC., Beaverton, OR, USA). Seebeck effect was exploited to verify conductivity type using a Keithley 6517A multimeter (TEKTRONIX, INC., Beaverton, OR, USA) and a soldering station [81].

## 3. Results

The described characterization techniques were used to characterize the structural, optical, and electrical properties of the doped ZnO layers.

### 3.1. Structural Characterization

**Figure 1:**
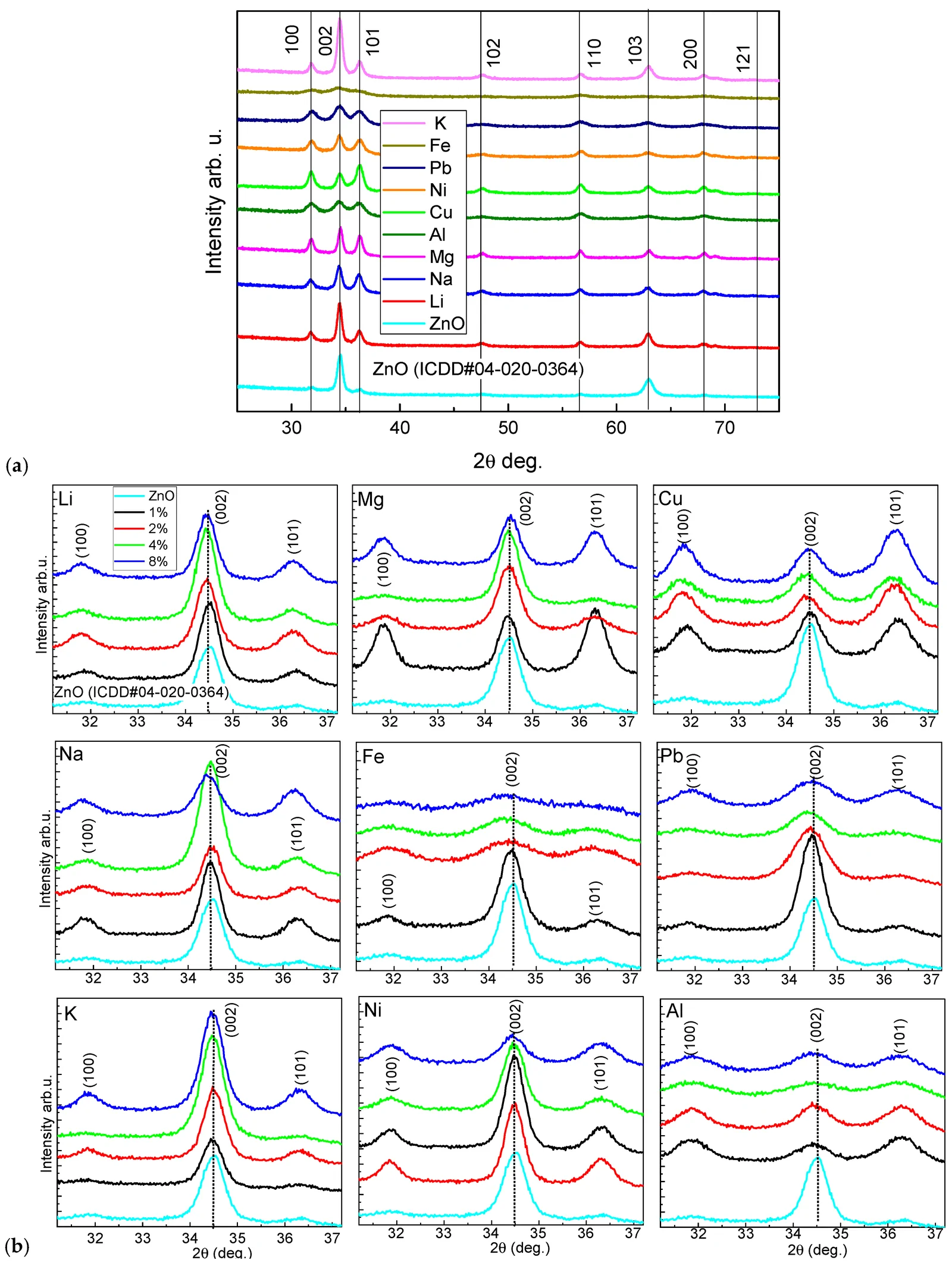
(**a**) X-ray diffraction patterns of undoped ZnO and ZnO thin films doped with 8 at% Na, Li, Fe, Cu, Mg, Ni, Pb, K, and Al. The indexed diffraction peaks correspond to the hexagonal wurtzite ZnO structure (ICDD No. 04-020-0364). (**b**) Enlarged views of the (100), (002), and (101) reflections illustrating the effect of the different dopant ions on the peak position and intensity. The diffraction patterns have been vertically shifted for clarity.

The crystal structure of the doped ZnO films was first investigated to determine whether replacement of Zn with different metal elements modifies the crystallinity or induces secondary phases. The X-ray diffraction (XRD) patterns of undoped ZnO and ZnO thin films doped with 8 at% of different metal ions are presented in Figure 1a, while enlarged views of the main diffraction peaks are shown in Figure 1b. All diffraction peaks can be indexed to the hexagonal wurtzite ZnO structure (ICDD No. 04-020-0364), confirming that the crystal structure is preserved after doping. No additional reflections corresponding to secondary phases or impurity oxides are detected, indicating either successful incorporation of the dopant ions into the ZnO lattice or concentrations below the XRD detection limit. The diffraction patterns exhibit the characteristic ZnO reflections indexed as (100), (002), (101), (102), (110), (103), (200), and (112). The (002) reflection remains the most intense peak for all samples, indicating a preferred orientation along the c-axis irrespective of the dopant species. Structural parameters calculated from the XRD data are summarized in Table 1 and Table 2. The average crystallite size, estimated from the FWHM of the dominant diffraction peak using the Halder–Wagner method, varies between 5.3 and 15.7 nm, demonstrating that the dopant species strongly influence crystallite growth. Although the crystallite size and peak broadening differ considerably among the doped films, the diffraction peak positions remain nearly unchanged. The absence of measurable peak shifts suggests that doping with different metal ions does not significantly alter the lattice parameters of ZnO at the investigated doping level. Instead, the primary effect of doping is reflected in changes in diffraction intensity and peak width, indicating differences in crystalline quality rather than in crystal structure [71,82]. 

Particularly, Fe, Pb and Al transition metal doping strongly broadens XRD peaks due to reduced crystallite sizes (see Table 2). Doping ZnO with elements like Fe, Pb, or Al reduces crystallite size because foreign atoms create lattice strain, introduce grain boundary pinning, and disrupt the orderly arrangement needed for the crystals to grow larger [83]. On the other hand, alkali metals (like Li, Na, K, Mg) provide larger ZnO crystallite grains because they promote oxygen vacancies, lower the activation energy for grain growth, and accelerate atomic diffusion [84].

To further quantify the effect of metal-ion incorporation on the ZnO lattice, the lattice parameters and microstrain were calculated from the XRD data using the Halder–Wagner method, and the results are summarized in Table 1. The a and c lattice parameters show only small variations among the investigated films, and the c/a ratio remains close to the characteristic value for wurtzite ZnO. This confirms that the average hexagonal ZnO structure is largely preserved upon metal-ion incorporation. In contrast, the microstrain varies more noticeably with dopant species and concentration, indicating differences in local lattice distortion. These results support the conclusion that doping mainly affects local structural disorder and crystallite growth rather than causing a significant change in the overall ZnO crystal structure.

**Figure 2:**
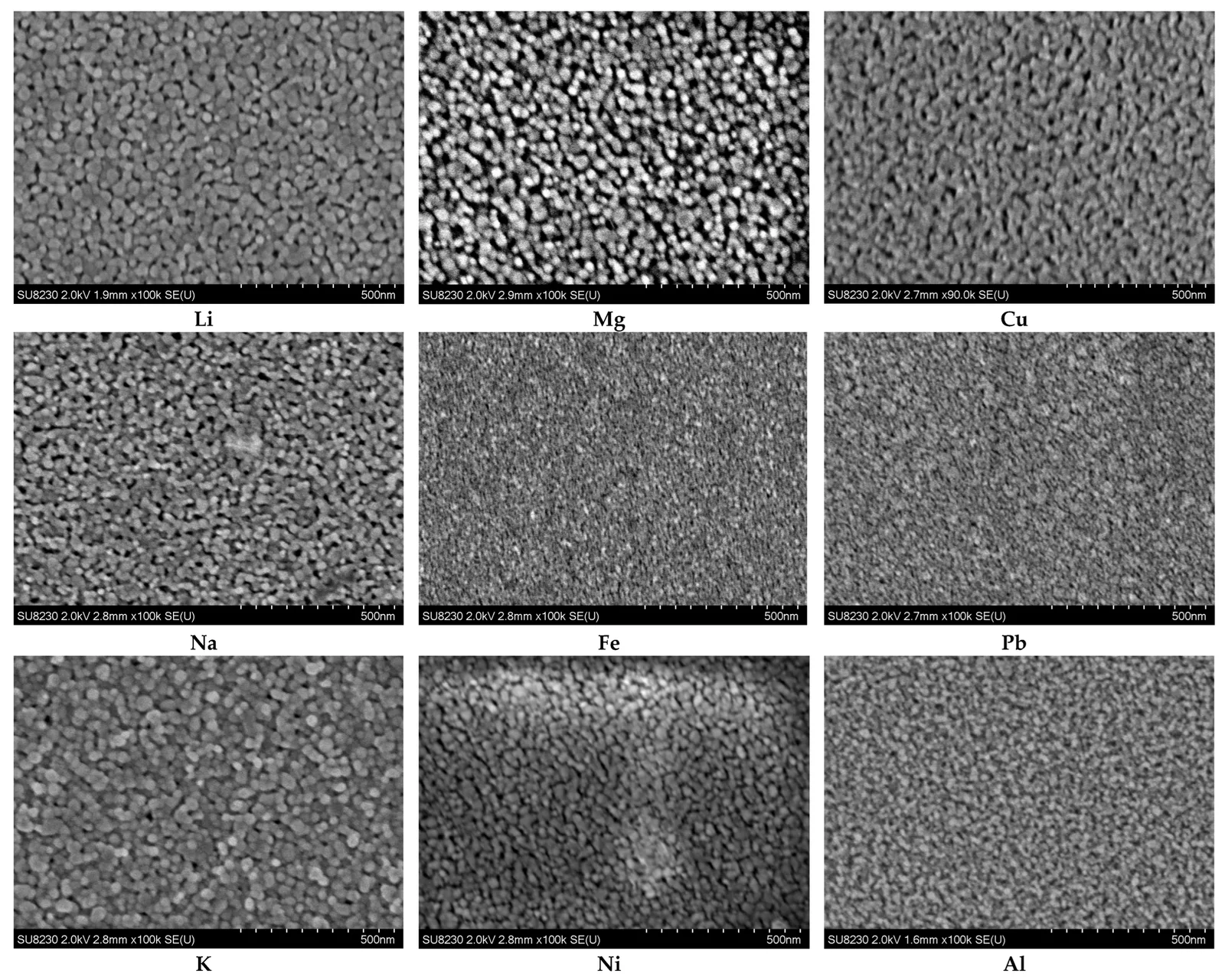
SEM surface morphology for different elements doped at 8%.

The influence of metal doping on the film morphology was examined by scanning electron microscopy. Representative surface images are presented in Figure 2. All films exhibit continuous surface coverage without visible cracks or delamination, demonstrating that the solution-based deposition method provides uniform thin films over the investigated compositional range. Although the average grain size varies among samples, no pronounced morphological degradation is observed even at the highest dopant concentration.

**Figure 3:**
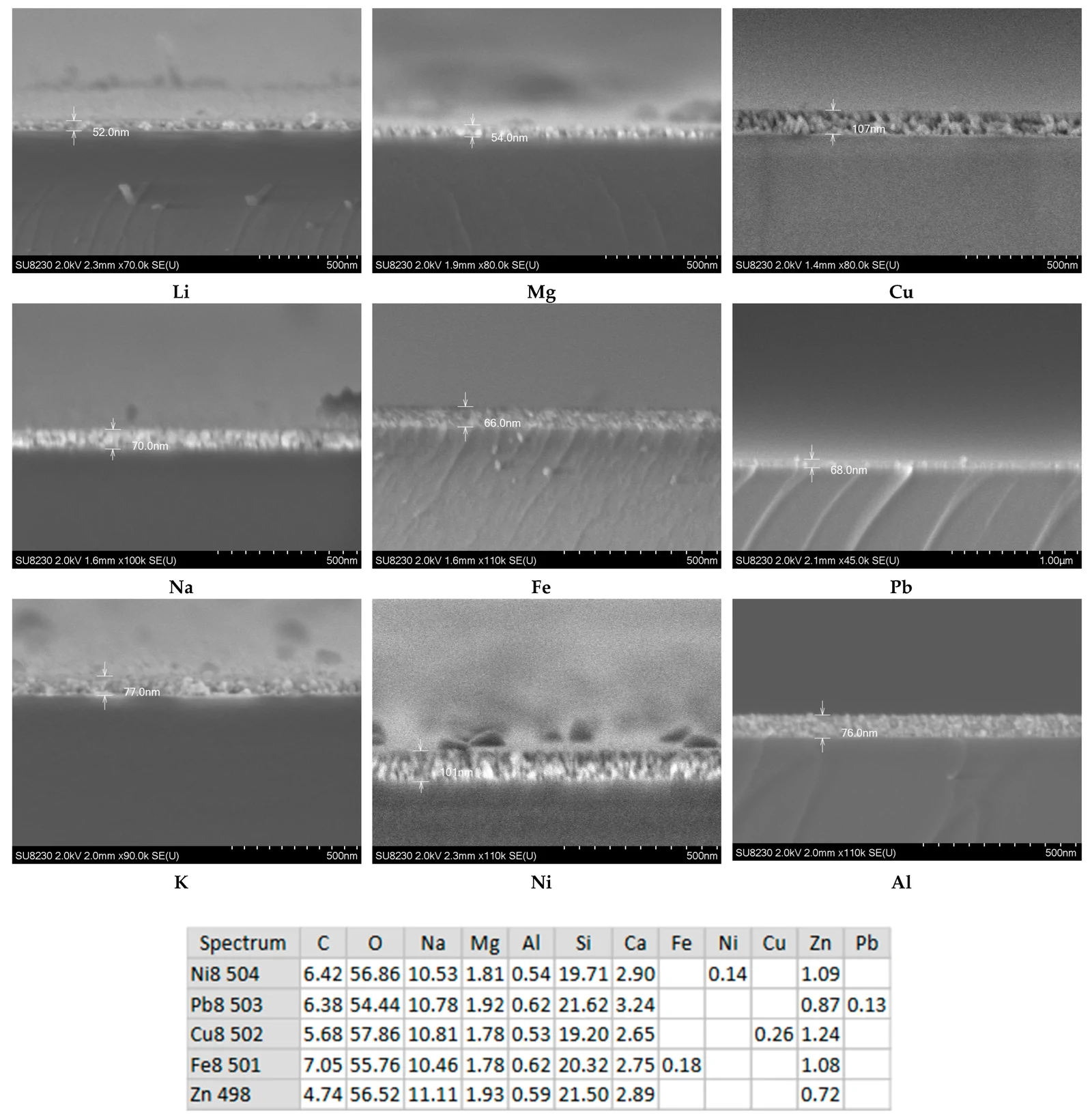
SEM cross-sections for different 8% doping levels and EDX measurements in selected samples.

Cross-sectional SEM images (Figure 3) reveal film thicknesses between approximately 70 and 100 nm for all investigated samples. Since identical precursor concentrations and deposition parameters were used throughout the study, only relatively small thickness variations were observed. Nevertheless, films containing transition-metal dopants such as Ni, Cu, and Al tend to be slightly thicker than those containing alkali metals. This trend suggests that doping precursors can modify the mixed precursor decomposition, crystallization kinetics, or solution viscosity during spin coating, thereby influencing the final film thickness.

Overall, the structural characterization demonstrates that metal doping modifies the microstructure of nanocrystalline ZnO without altering its crystalline phase, thus providing a common structural platform for subsequent comparison of the optical and electrical properties.

The EDX concentration (see Figure 3) is affected by the glass substrate (consisting of main elements O, Si, Na, Ca, Mg, Al, K [85]); thus, we were able to resolve only the highest 8% doping in the samples: Ni 8%, Pb 8%, Cu 8%, and Fe 8%. Using the EDX spectra fitting with Quantax Esprit 2.0 software (Quantification model P/B—ZAF, series fit), we determined the doping metal concentrations (conc. = [X]/([X] + [Zn])) of 11 ± 5%, 13 ± 5%, 17 ± 5%, and 14 ± 5%, respectively.

### 3.2. Optical Properties

#### 3.2.1. Optical Absorption and Excitonic Properties

**Figure 4:**
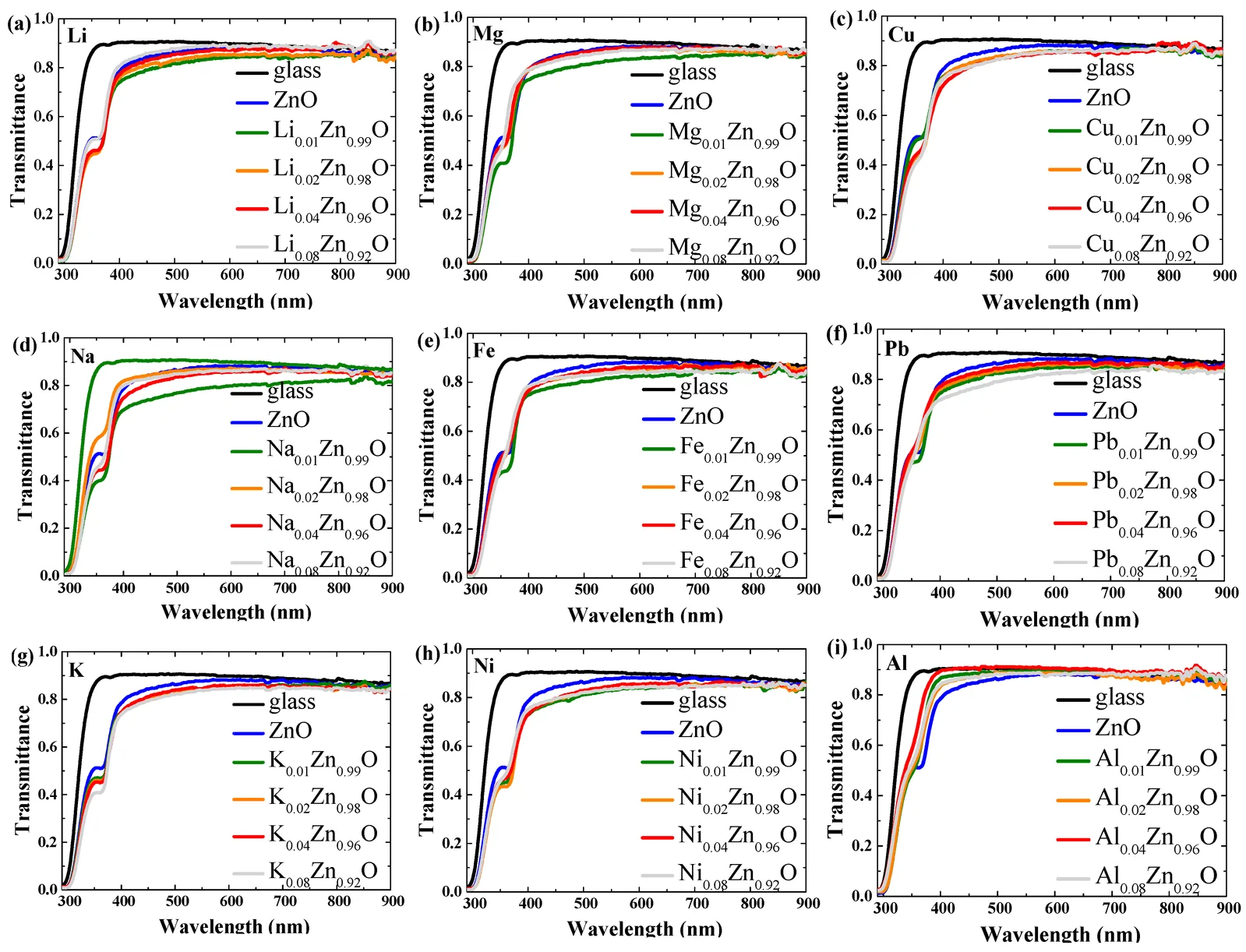
Transmission spectra of the ZnO samples doped with (**a**) Li, (**b**) Mg, (**c**) Cu, (**d**) Na, (**e**) Fe, (**f**) Pb, (**g**) K, (**h**) Ni, and (**i**) Al. The transmission of the glass substrate is shown for comparison.

The optical transmission spectra of the investigated ZnO films are presented in Figure 4. All samples exhibit high transparency throughout the visible spectral range, with transmittance exceeding 80% above the absorption edge, demonstrating that substitutional metal doping does not significantly compromise the optical transparency of solution-processed ZnO. This characteristic is essential for applications requiring transparent conductive or optoelectronic layers [86].

A pronounced absorption feature is observed near 370 nm in the pure ZnO film, corresponding to the free-exciton transition [87]. The preservation or suppression of this excitonic resonance provides valuable insights into the influence of different dopants on the electronic structure of ZnO. While Li- and Na-doped films retain a relatively sharp excitonic feature even at elevated dopant concentrations, the exciton peak becomes progressively broadened and eventually almost disappears in Fe-, Pb-, and Al-doped samples.

The suppression of excitonic absorption is consistent with increased free-carrier screening and enhanced structural disorder introduced by heavy doping [88]. Both mechanisms reduce the exciton binding probability and broaden the absorption edge. As shown below, the same samples also exhibit reduced photoluminescence efficiency, indicating that the degradation of excitonic absorption is accompanied by enhanced non-radiative recombination.

#### 3.2.2. Radiative Recombination

**Figure 5:**
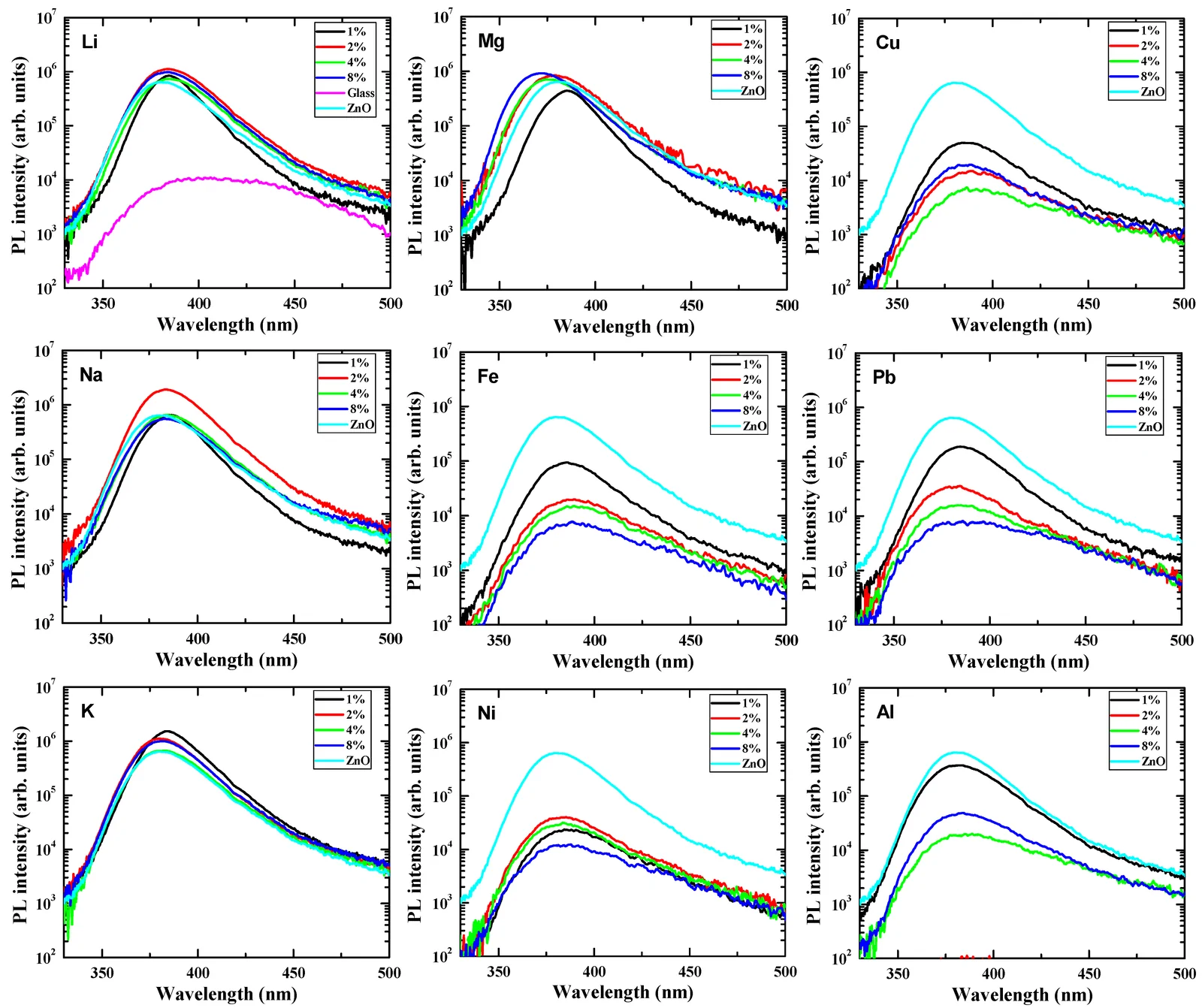
PL spectra at different doping levels at 0.1 mJ/cm^2^ excitation compared to ZnO emission. Glass emission from the sample backside is shown for comparison.

The photoluminescence spectra presented in Figure 5 reveal that metal doping modifies both the radiative efficiency and emission energy of nanocrystalline ZnO. The observed trends closely follow the changes identified in the transmission spectra, demonstrating that the same dopant-induced modifications to the electronic structure govern both optical absorption and light emission.

Three distinct categories of dopants can be identified. Alkali metals (Li, Na, and K) substantially enhance the near-band-edge emission compared with undoped ZnO, indicating the suppression of non-radiative recombination pathways and improved excitonic radiative efficiency. In contrast, transition metals (Fe, Ni, Cu, and Pb) strongly quench the luminescence, consistent with the formation of deep defect levels acting as efficient non-radiative recombination centers [89]. Magnesium exhibits a unique behavior, producing a pronounced blue shift in the emission while maintaining relatively high PL efficiency, reflecting the widening of the ZnO bandgap through Mg incorporation [90].

These observations are fully consistent with the transmission measurements. Samples exhibiting the strongest suppression of the excitonic absorption also show the lowest PL intensity, whereas films that preserve a well-defined excitonic absorption feature provide efficient radiative recombination. The excellent correlation between absorption and emission demonstrates that metal doping primarily modifies the density of electronic defects and the stability of excitonic states in nanocrystalline ZnO [91].

#### 3.2.3. Excitation-Dependent Photoluminescence and Amplified Spontaneous Emission

**Figure 6:**
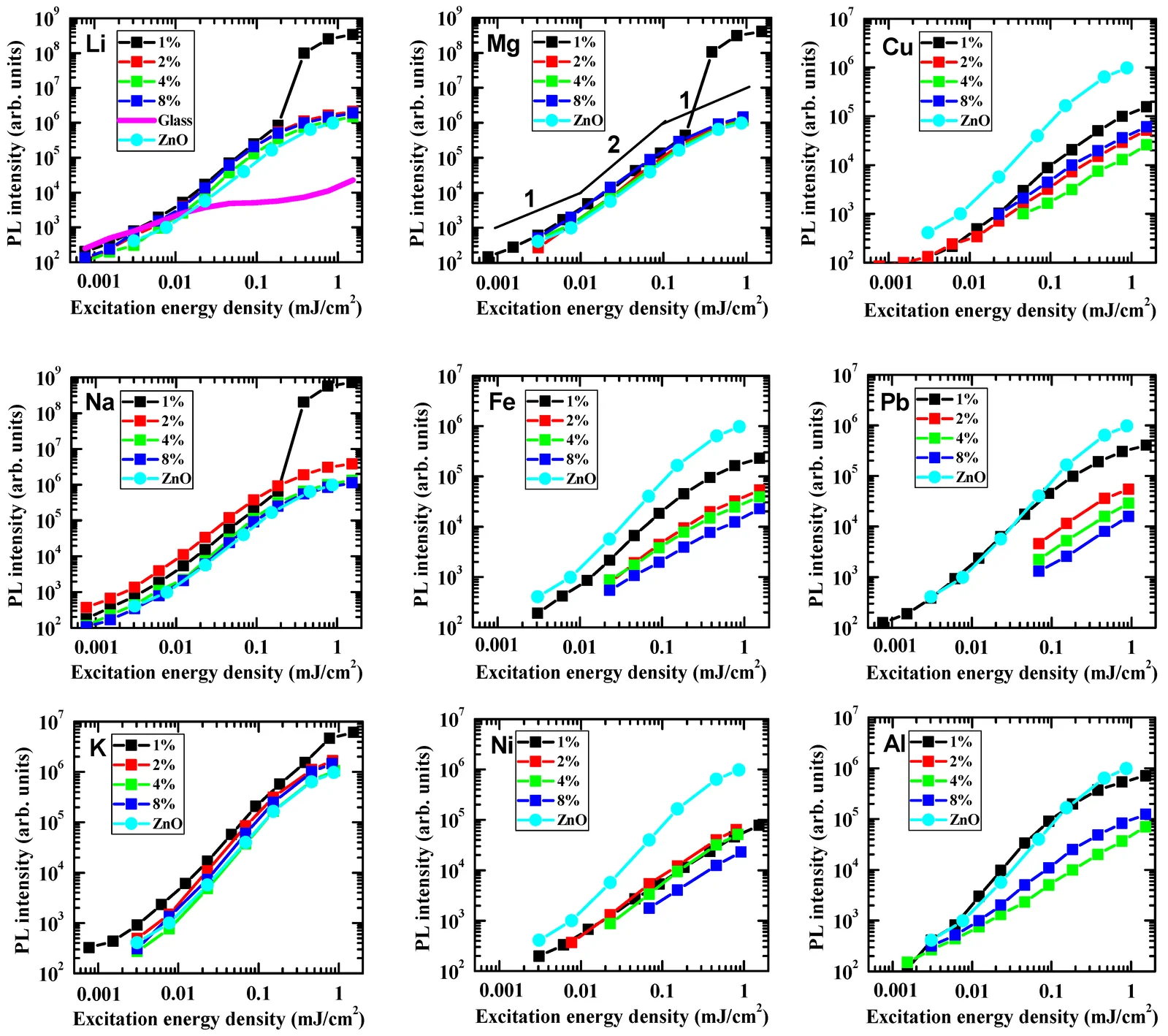
PL intensity versus excitation at different doping levels compared to ZnO. Lines show slopes of 1 and 2.

The excitation-fluence dependence of the photoluminescence provides further insight into the recombination mechanisms governing the optical response of the doped ZnO films. The integrated PL intensity as a function of excitation fluence is shown in Figure 6. All investigated samples exhibit characteristic power-law behavior, demonstrating that metal doping strongly modifies not only the emission efficiency but also the carrier recombination dynamics.

At low excitation fluences, the PL intensity increases almost linearly with excitation power (PL ~ P^1^) for most samples, indicating that the recombination process is dominated by defect-controlled carrier capture in the doped semiconductor [54]. As the excitation density increases, the slope gradually approaches two, reflecting the growing contribution of bimolecular electron-hole recombination and exciton formation after progressive saturation of non-radiative defect states. Similar behavior has been reported previously for ZnO and other wide-bandgap semiconductors [54].

A distinctly different behavior is observed in heavily Fe-, Cu-, Ni-, and Pb-doped films. In these samples, the PL intensity remains close to linear over a considerably wider excitation range, while the transition to the bimolecular recombination regime is significantly suppressed. Together with the strong quenching of the PL intensity and the disappearance of the excitonic absorption peak discussed above, this behavior indicates that these dopants introduce efficient defect-assisted non-radiative recombination pathways [91]. The increased density of defect states effectively competes with excitonic recombination, thereby reducing both the emission efficiency and the excitonic contribution to the optical response.

At the highest excitation fluences (>0.1 mJ/cm^2^), the slope decreases again in most samples. This behavior is attributed to progressive screening of Coulomb interactions at high carrier densities, resulting in exciton ionization and the onset of the Mott transition [92]. The exciton Mott density is 1.5 × 10^18^ cm^−3^ at 300 K in ZnO [93]. We use a 325 nm wavelength for excitation (with quantum energy *h*ν = 3.82 eV), for which the absorption coefficient α = 1.2 × 10^5^ cm^−1^. The average excited carrier density can be estimated as [94] *N_av_* = α*I*_0_/(2*h*ν) = 10^19^ cm^−3^ at *I*_0_ = 0.1 mJ/cm^2^ excitation fluence. *N_av_* exceeds the Mott density seven times. Therefore, the PL emission should be dominated by electron-hole plasma. Under varied excitation conditions, radiative recombination gradually changes from an excitonic process to recombination within an electron-hole plasma. This interpretation is consistent with both the weakening of the excitonic absorption feature observed in the transmission spectra and the excitation-induced shift in the PL peak discussed in the following section.

**Figure 7:**
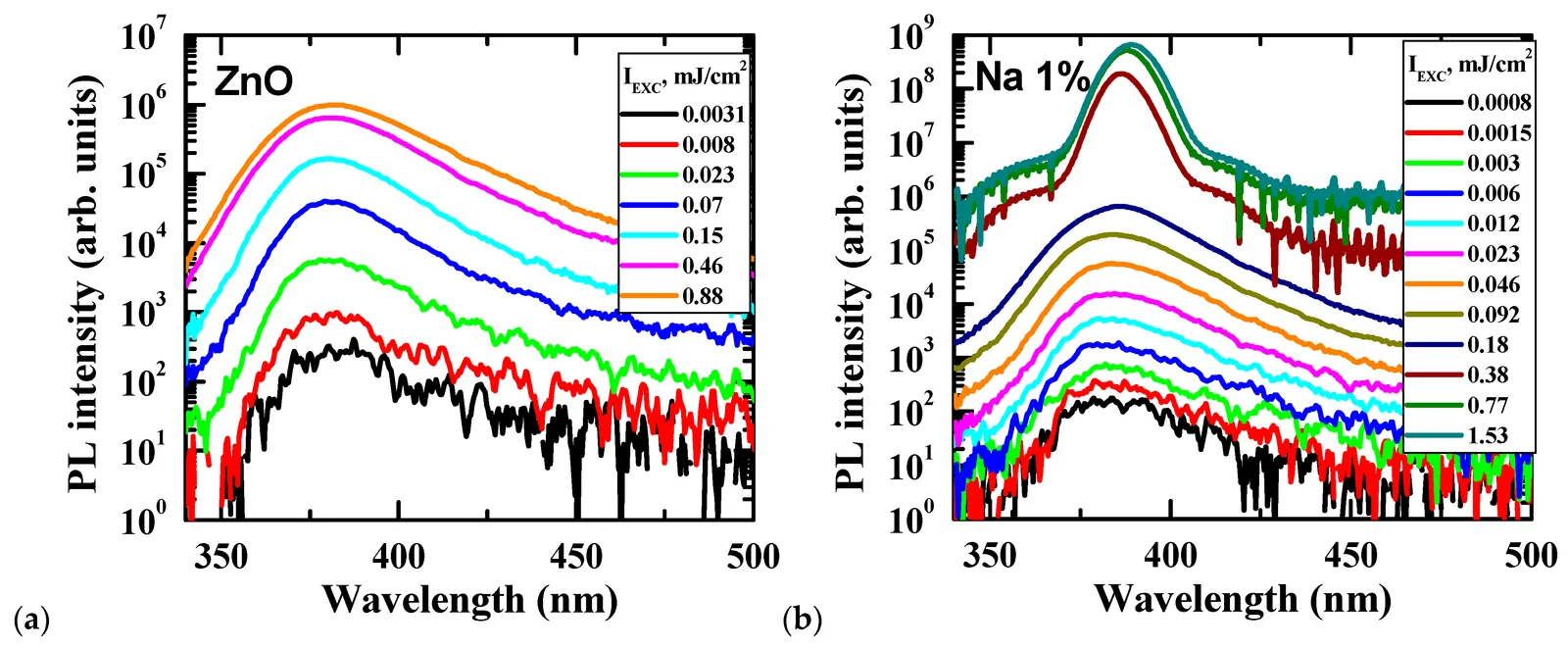
PL spectra vs. excitation in ZnO (**a**) and Na1% (**b**) samples. Appearance of a narrow and intense peak in the Na 1% sample corresponds to ASE. In the ASE regime, the spectra “wings” appear due to the streak camera intensifier halo effect for the ASE peak.

Moreover, Li-, Na-, and Mg-doped samples with 1% doping exhibit a rapid superlinear increase in PL intensity already at relatively low excitation fluences. Above the 0.21 ± 0.03 mJ cm^−2^ threshold for Li 1%, Na 1%, and Mg 1% samples, the emission spectra simultaneously undergo pronounced, abrupt spectral narrowing (from 27 ± 4 nm to 11 ± 1 nm FWHM), accompanied by a strong increase in peak intensity (Figure 7). These are characteristic signatures of amplified spontaneous emission (ASE). Corresponding to the ASE threshold, *N_av_* = 2 × 10^19^ cm^−3^ is larger than the density of states in the valence and conduction bands, leading to carrier plasma degeneracy needed for stimulated emission [94]. The observed ASE threshold is comparable to previously reported values for ZnO thin films prepared by pulsed laser deposition [95], confirming that the solution-processed films investigated in this work possess optical quality sufficient for stimulated emission. The position of the ASE peak at 387 nm in our samples also agrees well with the literature value of 389 nm [96].

Overall, the excitation-dependent measurements demonstrate that metal doping modifies not only the efficiency of radiative recombination but also the sequence of carrier relaxation processes, which lead from defect-assisted recombination to excitonic emission, amplified spontaneous emission [96], and finally the high-density electron-hole plasma regime.

#### 3.2.4. Excitation-Dependent Evolution of Emission Energy

**Figure 8:**
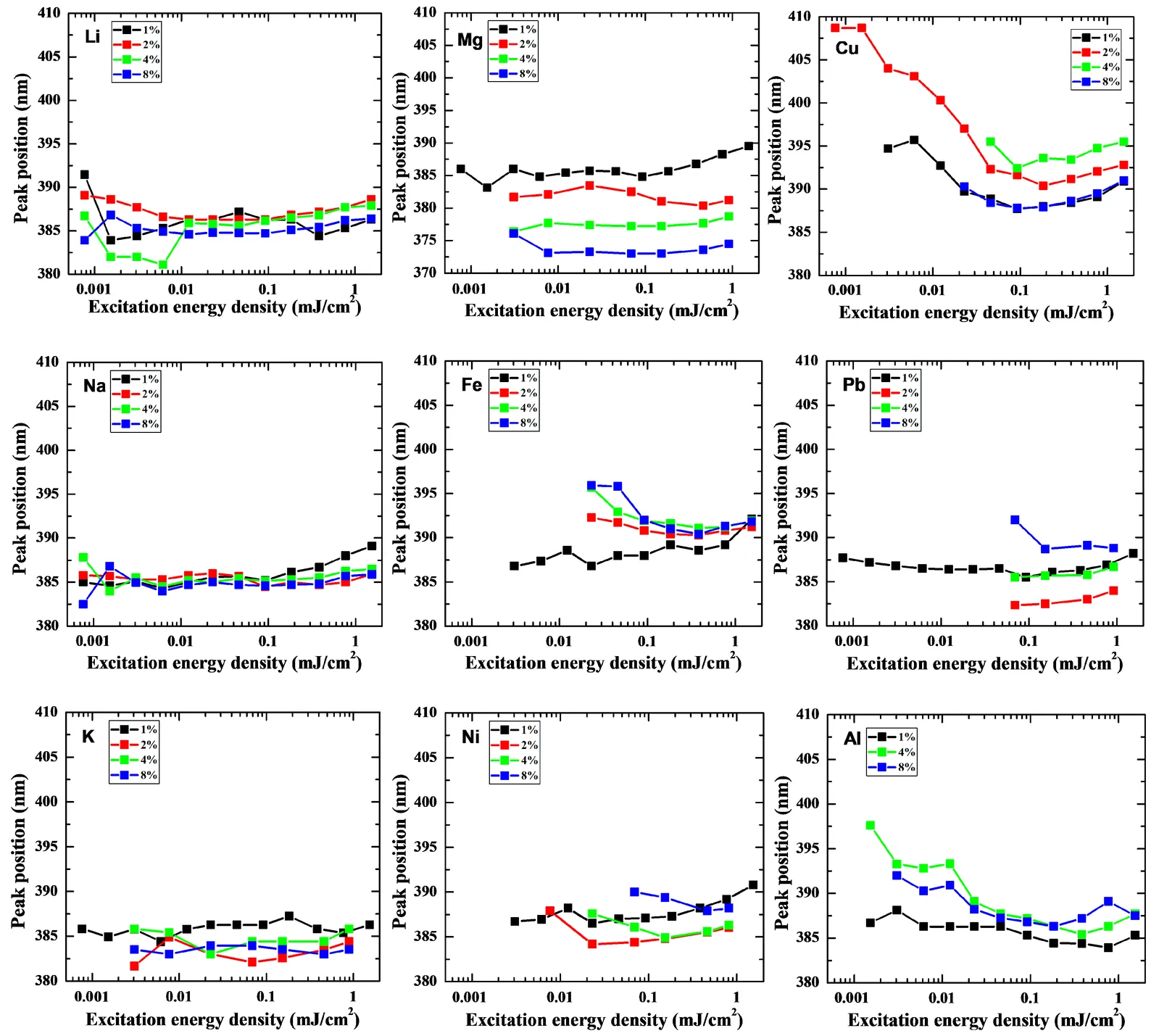
PL peak position vs. excitation for different elemental dopants.

The excitation-fluence dependence of the PL peak position provides additional insight into the evolution of the dominant radiative recombination mechanism in the investigated ZnO films. The emission peak wavelengths extracted from the spectra are summarized in Figure 8. Compared with the pronounced variations in PL intensity, the emission energy exhibits only relatively small changes with excitation fluence, indicating that the near-band-edge emission remains the dominant radiative channel throughout the investigated excitation range.

At low excitation densities, several samples exhibit a slight redshift of the emission peak. This behavior is particularly pronounced for Cu- and Fe-doped films, where the near-band-edge emission is substantially weakened. Under these conditions, defect-assisted recombination and the residual luminescence originating from the glass substrate contribute more significantly to the measured spectra, resulting in an apparent shift in the emission maximum toward longer wavelengths.

As the excitation fluence increases, the influence of defect-related emission gradually decreases owing to the saturation of non-radiative recombination centers, while the near-band-edge emission becomes progressively dominant. Consequently, the emission peak remains nearly constant over a broad excitation range for most samples, confirming that radiative recombination is primarily governed by excitonic transitions rather than deep-level emission.

At the highest excitation fluences, a slight red shift is again observed in most samples. This behavior is attributed to the increasing carrier density, which progressively screens the Coulomb interaction responsible for exciton formation. As the system approaches the Mott transition, radiative recombination gradually evolves from bound excitons to an electron-hole plasma, producing the observed shift in the emission maximum [88]. This interpretation is fully consistent with the excitation-dependent PL intensity (Figure 6), the appearance of amplified spontaneous emission (Figure 7), and the gradual suppression of the excitonic absorption feature observed in the transmission spectra.

Among all investigated dopants, Mg exhibits distinctly different behavior. Increasing Mg concentration systematically shifts the emission toward shorter wavelengths while largely preserving the excitation dependence observed for undoped ZnO. This blue shift is consistent with the well-established widening of the ZnO bandgap following doping of Zn^2+^ with Mg^2+^, demonstrating that Mg predominantly modifies the intrinsic electronic structure rather than increasing the density of non-radiative recombination centers. In contrast, transition-metal dopants, particularly Fe and Cu, produce a pronounced red shift accompanied by strong PL quenching. The simultaneous reduction in emission intensity and shift toward longer wavelengths suggests that the observed spectral changes originate primarily from defect-assisted recombination and carrier localization rather than from a genuine reduction in the ZnO bandgap.

Overall, the excitation-dependent evolution of the PL peak demonstrates that metal doping influences the emission energy through two distinct mechanisms: intrinsic band-structure modification, as represented by Mg replacement, and defect-induced localization effects, which dominate in transition-metal-doped ZnO.

### 3.3. Electrical Properties

Electrical characterization provides direct information on the influence of metal doping on the equilibrium carrier concentration and transport properties of ZnO, thereby complementing the optical investigations presented above. While optical spectroscopy probes the recombination dynamics of photoexcited carriers, electrical measurements reveal how different dopants modify the intrinsic conductivity and conductivity type of the material. Together, these techniques enable a comprehensive assessment of the electronic functionality of the investigated ZnO films.

**Figure 9:**
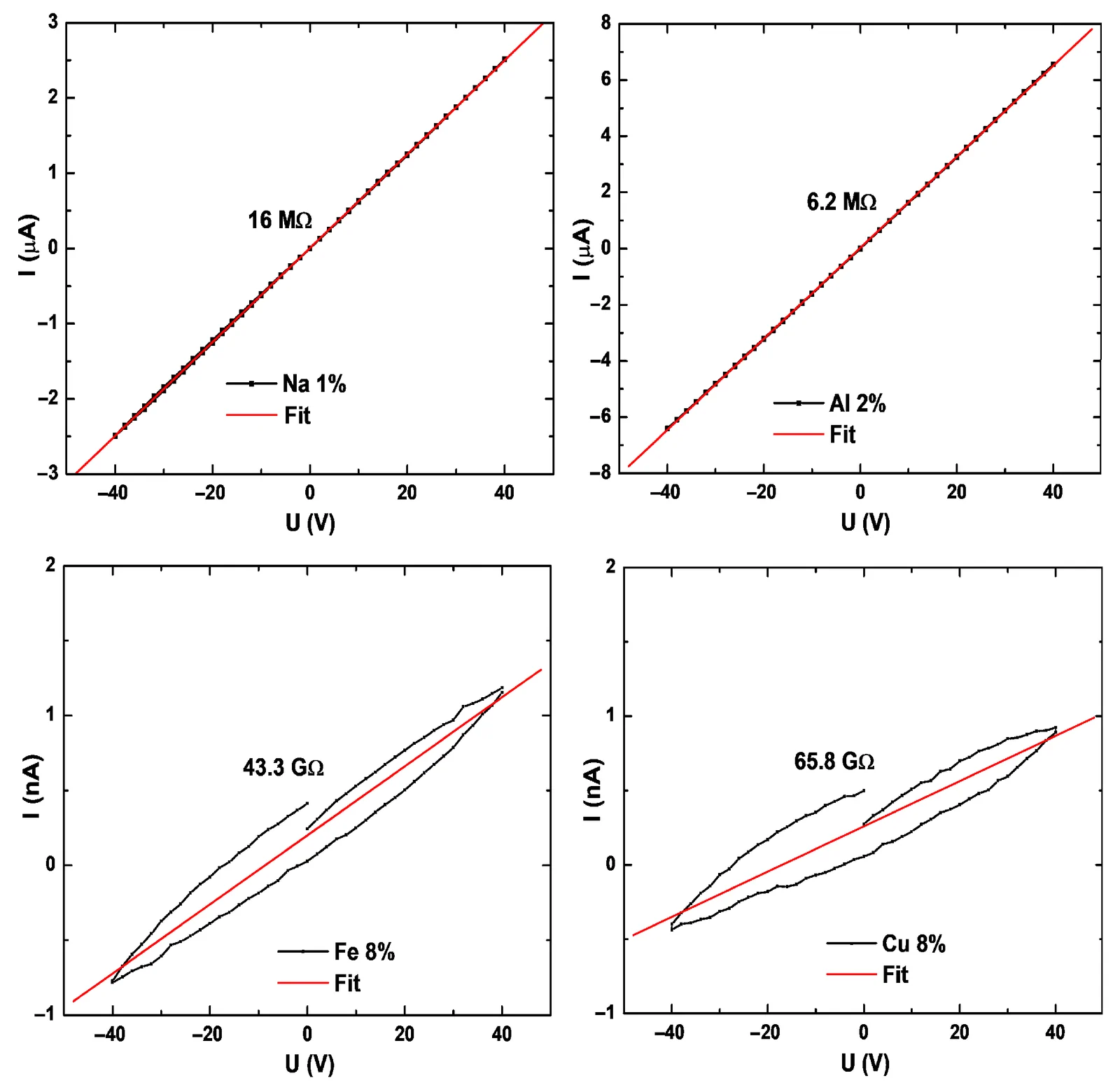
IV curves of selected samples. Linear fitted curves are used for resistivity determination. The resistivity values are indicated on the plots.

Current–voltage characteristics recorded for the investigated samples are presented in Figure 9. All measured structures exhibit nearly linear I–V characteristics, confirming the formation of good Ohmic contacts between the evaporated silver electrodes and the ZnO layers. High-resistivity samples show hysteresis due to large RC constants and possible carrier accumulation at defects. Consequently, the electrical conductivity could be reliably extracted from the measured resistance without significant influence of contact barriers (Table 2).

The electrical properties of ZnO are strongly influenced by the type and concentration of dopant, the associated defect chemistry, and the deposition and post-annealing conditions. Depending on these factors, doped ZnO may retain its intrinsic *n*-type character, exhibit enhanced or suppressed *n*-type conductivity, or undergo conversion to *p*-type conductivity.

Among alkali-metal dopants, Li can introduce acceptor-related defect levels in ZnO, although Li-doped ZnO often remains *n*-type because of native donor defects. Shinde et al. reported *n*-type conductivity in Li-doped ZnO films when the Li concentration was increased from 0.25 to 1%. The conductivity increased from 208 to 589 Ω^−1^cm^−1^. The remaining *n*-type behavior was attributed to native donor defects, particularly oxygen vacancies and zinc interstitials [97]. In another study, Li-doped ZnO thin films exhibited a conductivity of 1.12 × 10^−1^ Ω^−1^cm^−1^ [98].

Na doping has also been reported to produce both *n*-type and *p*-type ZnO, depending on the dopant concentrations. Na-doped ZnO films with Na/Zn molar ratios of 0.01, 0.02, 0.03, and 0.04 were prepared using the sol–gel spin-coating method. Films containing lower Na concentrations exhibited *n*-type conduction, with resistivities between 1.89 × 10^3^ and 2.35 × 10^3^ Ω cm. At the highest Na concentration, the films exhibited *p*-type conduction, with resistivities between 9.88 × 10^2^ and 5.41 × 10^2^ Ω cm [99].

A similar concentration- and temperature-dependent conversion was observed in K-doped ZnO. Films containing 1 wt.% K exhibited *n*-type conductivity after annealing at both 250 and 500 °C. Increasing the K content to 2 wt.% converted the conductivity to *p*-type at both annealing temperatures. For 3 wt.% K-doped ZnO, *p*-type behavior was observed only after annealing at 250 °C. The reported resistivities for ZnO:1%K annealed at 250 and 500 °C were 1.906 × 10^4^ and 1.212 × 10^3^ Ω·cm, respectively. The corresponding values for ZnO:2%K were 3.539 × 10^2^ and 4.244 × 10^3^ Ω·cm, while those for ZnO:3%K were 3.960 × 10^4^ and 3.049 × 10^2^ Ω·cm [100]. These observations demonstrate that alkali-metal doping does not automatically produce *p*-type ZnO, because the resulting carrier type depends on dopant incorporation, native defects, and thermal treatment.

Transition-metal doping generally modifies the native *n*-type conductivity of ZnO through carrier trapping, compensation, and defect formation. Co-doped ZnO samples showed conductivities ranging from 1.6 × 10^−7^ to 6.0 × 10^−8^ Ω^−1^cm^−1^. The material remained *n*-type, but the electron concentration decreased with increasing Co content because Co-related defects trapped or compensated donor-generated electrons. Consequently, the resistivity increased as the Co concentration increased [101].

Uzar et al. prepared undoped ZnO, Ni-doped ZnO, and Ni-B-codoped ZnO using a solution-processing technique. The reported conductivities were 0.085, 0.71, and 1.1 Ω^−1^cm^−1^, respectively, and all samples exhibited *n*-type semiconducting behavior [102]. In contrast, ZnO films containing 3 and 6% Cu exhibited *p*-type behavior. Increasing the Cu concentration increased the hole concentration and increased the conductivity from 0.006 to 0.012 Ω^−1^cm^−1^ [103].

The electrical behavior of Al-doped ZnO also depends strongly on the dopant configuration and processing conditions. Al doping alone commonly produces *n*-type conduction because Al^3+^ substitutes for Zn^2+^ and contributes additional electrons. Al-doped ZnO samples were reported to exhibit *n*-type conductivity of approximately 10^−1^ Ω^−1^cm^−1^ [104].

In contrast to Al-only doping, Kalyanaraman et al. produced *p*-type ZnO through Al and N codoping. ZnO films containing 1 and 2 mol.% Al exhibited conductivities of approximately 19 and 12 Ω^−1^cm^−1^, respectively. The *p*-type behavior was attributed to the combined effect of Al and N incorporation rather than to Al doping alone [105].

Other donor-type dopants generally preserve or enhance the *n*-type character of ZnO. ZnO films containing 6% Mg exhibited *n*-type conduction, with a resistivity of 3.07 Ω·cm [106].

Overall, the reported results demonstrate that the electrical properties of doped ZnO cannot be predicted solely from the nominal valence of the dopant. Alkali-metal and acceptor dopants may produce *p*-type conductivity at suitable concentrations and processing conditions, but low dopant concentrations frequently retain *n*-type behavior because of native oxygen vacancies, zinc interstitials, and other donor defects. Trivalent and pentavalent dopants, such as Al, La, Dy, and Nb, generally promote *n*-type conductivity by supplying additional electrons. Transition-metal dopants may either compensate native donors, as observed for Co, or promote *p*-type behavior under specific conditions, as reported for Cu and Mn. These literature values provide a broad reference for evaluating the conductivity and conduction type observed in the present samples.

The cumulative properties of differently doped ZnO layers are summarized in Table 2 and are further discussed in detail in the next section.

**Table 2:** Properties of ZnO layers with different dopants.

Sample	Doping (%)	Crystallite Size (nm)	Thickness (nm)	PL Peak * (nm)	PL Intensity * (rel. to ZnO)	Type	Conductivity 10^−3^ (Ω^−1^cm^−1^)	Conductivity 10^−3^ (Ω^−1^cm^−1^)
ZnO	0	8.9 (±1.1)	50	382.3	1	*n*	508	*n* 9.8 [65]; *n* 85 [101]
Li	1	11.1 (±0.7)		386.3	3.5	*n*	12.5	*n* 4.8 (0.25%) [97]
	2	10.8 (±0.4)		386.3	2.7	*n*	6.2	*n* 1.7 (1%) [97]
	4	12.4 (±0.5)		386.2	1.8	*n*	2.9	*p* 15.6 (5%) [65]
	8	10.1 (±0.7)	52	384.7	2.8	*p*	4.4	*p* 20 (10%) [65]
Na	1	9.4 (±0.4)		385.2	2.9	*n*	12.4	*n* 0.53 (1%) [99]
	2	12.8 (±0.5)		384.5	5.4	*n*	0.07	*n* 0.43 (2%) [99]
	4	11.4 (±0.8)		385.1	1.6	*p*	0.24	*p* 1.01 (3%) [99]
	8	8.2 (±1.2)	70	384.6	1.3	*n*	0.59	*p* 1.85 (4%) [99]
K	1	9.4 (±0.5)		386.3	3.1	*n*	2.08	*p* 0.83 (1%) [100]
	2	14.8 (±0.7)		382.2	2.1	*n*	0.95	*p* 3.3 (3%) [100]
	4	15.7 (±1.0)		384.4	1.0	*n*	0.41	
	8	13.2 (±0.6)	77	384.0	1.6	*p*	0.048	
Mg	1	7.4 (±0.5)		384.8	2.0	*n*	0.11	*n* 0.86 (1%) [68]
	2	9.4 (±0.7)		382.5	1.4	*n*	1.54	*n* 0.019 (2%) [68]
	4	9.8 (±1.0)		377.2	1.1	*n*	3.28	*n* 0.015 (3%) [68]
	8	11.7 (±0.6)	54	373.0	2.0	*n*	4.1	*n* 325 (6%) [100]
Fe	1	9.4 (±0.5)		388.0	0.28	*n*	0.45	*n* 120 (2%) [67]
	2	5.9 (±0.4)		390.8	0.068	*n*	0.05	*n* 40 (3%) [67]
	4	4.7 (±1.2)		391.6	0.055	*n*	0.011	*p* 20 (4%) [67]
	8	3.4 (±0.6)	66	392.0	0.028	*n*	0.0056	
Ni	1	8.4 (±0.5)		387.1	0.079	*n*	0.27	*n* 0.85 (2%) [66]
	2	9.6 (±0.6)		384.4	0.10	*p*	0.11	*n* 0.83 (4%) [66]
	4	10.1 (±1.0)		386.1	0.074	*n*	0.14	*n* 0.83 (6%) [66]
	8	6.6 (±0.3)	101	390.0	0.036	*n*	0.022	*n* 0.83 (8%) [66]
Cu	1	8.4 (±0.5)		387.7	0.13	*p*	0.0086	*p* 3 (3%) [103]
	2	7.7 (±0.7)		391.6	0.046	*n*	0.0054	*p* 12 (6%) [103]
	4	7.9 (±1.1)		392.4	0.022	*n*	0.00304	*n* 0.1 (4%) [70]
	8	6.4 (±0.6); CuO—8.9 (±0.6)	107	387.8	0.065	*n*	0.00304	
Pb	1	9.4 (±0.5)		385.5	0.65	*n*	0.44	*n* 10^−3^ (1%) [69]
	2	10.1 (±0.8)		382.2	0.091	*n*	0.071	*n* 10^−6^ (3%) [69]
	4	7.3 (±0.2)		385.5	0.045	*n*	0.0015	*n* 10^−6^ (5%) [69]
	8	6.1 (±0.5)	68	392.0	0.024	*n*	0.0032	*n* 10^−5^ (10%) [69]
Al	1	8.4 (±0.5)		385.3	1.3	*n*	0.27	*p* 19 × 10^3^ (1%) [105]
	2	7.6 (±0.3)				*n*	32.3	*p* 12 × 10^3^ (2%) [105]
	4	4.1 (±0.4)		387.2	0.072	*n*	0.11	
	8	5.4 (±0.6)	76	386.7	0.16	*n*	12.5	

* at 0.1 mJ/cm^2^ excitation. Red font color indicates increased grain size (PL wavelength), while green indicates a reduced one, with respect to ZnO.

## 4. Discussion

The present study demonstrates that metal doping provides an effective strategy for tailoring the structural, optical, and electrical properties of solution-processed nanocrystalline ZnO. Since all investigated films were synthesized using an identical precursor chemistry, deposition process, and annealing conditions, the observed differences can be attributed primarily to the intrinsic influence of the incorporated dopants rather than to variations arising from the fabrication procedure. Such a systematic comparison enables direct evaluation of the role of different metal dopants in determining the physical properties of nanocrystalline ZnO.

Although the investigated dopants produce only moderate changes in the crystal structure, they induce substantial modifications of the optical and electrical response. The XRD measurements confirm that all films retain the hexagonal wurtzite ZnO crystal structure without detectable secondary crystalline phases, while the crystallite size varies between approximately 5 and 15 nm depending on the incorporated dopant. The relatively small structural variations, compared with the much larger changes observed in the optical and electrical properties, indicate that the dominant mechanisms governing the material’s performance are electronic rather than structural. Consequently, the primary role of metal doping is not to modify the crystal phase but to alter the electronic structure, carrier concentration, and defect population of ZnO.

The optical characterization reveals a strong correlation between the absorption and emission properties of the investigated films. The undoped ZnO exhibits a pronounced excitonic absorption feature near the band edge together with efficient near-band-edge photoluminescence. In contrast, transition-metal doping progressively suppresses both the excitonic absorption and the PL intensity, whereas alkali-metal doping largely preserves the excitonic absorption while enhancing the radiative emission. These observations demonstrate that optical absorption and photoluminescence originate from the same underlying electronic structure and, therefore, should not be interpreted independently. Instead, both measurements consistently indicate how metal doping influences the stability of excitonic states and the competition between radiative and non-radiative recombination.

The excitation-dependent photoluminescence measurements provide further insight into the evolution of carrier recombination with increasing carrier density. At low excitation fluences, the nearly linear dependence of PL intensity on excitation power indicates that carrier recombination is strongly influenced by defect-assisted processes. As the excitation density increases, the recombination gradually becomes dominated by bimolecular radiative processes, resulting in a superlinear increase in the emission intensity. For Li-, Na-, and Mg-doped samples, this transition is followed by the appearance of amplified spontaneous emission, demonstrating that these materials possess sufficiently low optical losses and high optical gain to support stimulated emission. At the highest excitation densities, the reduced power-law slope together with the excitation-dependent spectral evolution is consistent with progressive exciton screening and the onset of the Mott transition, where radiative recombination gradually evolves from bound excitons toward an electron-hole plasma.

An important observation emerging from the excitation-dependent measurements is that the investigated dopants do not introduce fundamentally different recombination mechanisms. Instead, they primarily modify the excitation density at which transitions between defect-assisted recombination, excitonic recombination, amplified spontaneous emission, and high-density carrier recombination occur. Consequently, metal doping acts as a control parameter that shifts the boundaries between different recombination regimes rather than changing the sequence of physical processes governing carrier relaxation.

Among the investigated dopants, alkali metals exhibit particularly interesting behavior. Low concentrations of Li and Na significantly increase electrical conductivity while simultaneously enhancing near-band-edge photoluminescence. These observations indicate efficient donor activation accompanied by relatively limited formation of non-radiative recombination centers. Lithium is especially remarkable because its electrical behavior strongly depends on concentration. While low Li concentrations produce highly conductive *n*-type ZnO, increasing the Li content results in conductivity inversion and stable *p*-type conduction. This amphoteric behavior has been widely discussed in the literature and is generally attributed to the coexistence of interstitial Li donors and substitutional Li acceptors [107]. The possibility of controlling both conductivity magnitude and conductivity type using the same dopant is particularly attractive for future ZnO homojunction devices and transparent electronic applications [108].

Magnesium exhibits a distinctly different behavior from the remaining investigated dopants. Unlike transition metals, Mg does not strongly quench the photoluminescence, but instead systematically shifts both the absorption edge and the emission wavelength toward shorter wavelengths. This behavior is consistent with the formation of a ZnMgO alloy and the corresponding widening of the bandgap. The preservation of relatively efficient radiative recombination indicates that Mg introduces comparatively few non-radiative recombination centers, making it particularly suitable for bandgap engineering and wavelength tuning of ZnO-based ultraviolet optoelectronic devices.

In contrast, Fe, Ni, Cu, and Pb exhibit remarkably similar optical and electrical behavior despite their different chemical nature. These dopants simultaneously reduce the electrical conductivity, suppress the excitonic absorption feature, and strongly quench the near-band-edge photoluminescence. The excellent agreement between these independent measurements strongly suggests that transition-metal doping predominantly introduces defect states acting as efficient non-radiative recombination and carrier-compensation centers. Doping ZnO with elements like Fe, Ni, Cu, and Pb increases disorder and strain, and promotes the formation of oxygen vacancies [109,110], which are deep donors that reduce conductivity [111] and effectively trap electrons [112]. Consequently, the observed reduction in PL intensity cannot be attributed solely to changes in the band structure but rather reflects the increasing density of electrically active defects that compete with radiative recombination. The simultaneous decrease in conductivity further indicates that these vacancy defects effectively trap or compensate free carriers, thereby reducing both electrical transport and photoluminescence efficiency.

The correlation between the optical and electrical properties represents one of the most significant findings of the present work. Samples exhibiting the strongest photoluminescence quenching consistently display the lowest electrical conductivity and the most pronounced suppression of excitonic absorption. Conversely, films maintaining efficient near-band-edge emission generally exhibit relatively high conductivity and well-defined excitonic absorption features. Such excellent agreement among three independent characterization techniques demonstrates that the optical and electrical properties of nanocrystalline ZnO are governed by the same underlying electronic processes, namely the concentration of free carriers and the density of defect-related electronic states.

Based on the experimental observations, the investigated dopants can be classified according to three dominant mechanisms governing the physical properties of ZnO. The first mechanism is carrier-density engineering, represented primarily by Li, Na, and Al, which modifies the equilibrium carrier concentration and consequently influences both electrical conductivity and excitonic screening. The second mechanism is defect engineering, characteristic of Fe, Ni, Cu, and Pb, where the dominant effect is the introduction of non-radiative recombination centers [113] and carrier compensation [114]. Finally, band-structure engineering, represented by Mg substitution, primarily modifies the intrinsic electronic structure through bandgap widening while largely preserving efficient radiative recombination [114]. This classification provides a unified interpretation of the structural, optical, and electrical measurements and explains the systematic trends observed throughout the present study.

From an application perspective, the results demonstrate that solution-processed ZnO can be suggested for different optoelectronic functions through appropriate selection of dopants [115]. Alkali-metal doping is particularly promising for ultraviolet emitters because of the enhanced near-band-edge emission and the observation of amplified spontaneous emission. Magnesium offers an efficient route for tuning the emission wavelength while maintaining good optical quality, making it attractive for ultraviolet photonic devices. Aluminum provides highly conductive *n*-type ZnO, which may be suitable for transparent electrodes, whereas transition-metal doping produces highly compensated and resistive ZnO that may be advantageous for electrically insulating layers or carrier-blocking structures [116]. The systematic comparison presented here therefore proposes basic guidelines for selecting dopants according to the intended device application.

## 5. Conclusions

In this work, we systematically investigated the influence of metal doping on the structural, optical, and electrical properties of solution-processed nanocrystalline ZnO thin films prepared by an identical spin-coating route. The use of a common synthesis procedure for all investigated dopants enabled direct comparison of their intrinsic effects, while minimizing variations associated with different fabrication methods.

Although the crystal structure remained largely unchanged for all investigated compositions, metal doping induced pronounced modifications in the optical and electrical properties. The experimental results demonstrate that these changes originate primarily from electronic rather than structural effects, highlighting the dominant role of carrier concentration and defect states in determining the functionality of nanocrystalline ZnO.

A comprehensive analysis of the structural, optical, and electrical measurements revealed that the investigated dopants modify ZnO through four principal mechanisms with plausible applications: (1) carrier-density engineering, represented by Li, Na, and Al, enabling efficient control of electrical conductivity and conductivity type for *p*- or *n*-type contacts in optoelectronic devices such as solar cells, detectors, and transistors; (2) optical gain engineering, represented by Li, Na, and Mg, enabling efficient UV ZnO lasers; (3) defect engineering, represented by Fe, Ni, Cu, and Pb, introducing carrier-compensation and non-radiative recombination centers that simultaneously suppress photoluminescence and electrical conductivity, providing high resistivity layers for isolation regions in high-frequency and high-power electronics; and (4) band-structure engineering, represented by Mg replacement, predominantly widening the ZnO bandgap while preserving efficient near-band-edge emission, allowing production of ZnO/MgZnO quantum well emitters and detectors.

One of the most significant findings of this work is the strong correlation observed between optical absorption, photoluminescence, and electrical conductivity. Samples exhibiting efficient excitonic absorption consistently show strong near-band-edge emission and relatively high electrical conductivity, whereas transition-metal-doped films simultaneously display suppression of excitonic absorption, photoluminescence quenching, and reduced conductivity. This correlation demonstrates that the optical and electrical properties of nanocrystalline ZnO are governed by the same fundamental electronic processes, namely the balance between free carriers, excitonic recombination, and defect-assisted non-radiative relaxation.

The excitation-dependent photoluminescence measurements further demonstrate that metal doping does not fundamentally alter the sequence of carrier recombination processes, but instead shifts the excitation density at which the material evolves from defect-assisted recombination to excitonic emission, amplified spontaneous emission, and finally, the high-density electron-hole plasma regime. This finding provides new insight into the role of doping metals in controlling carrier dynamics in ZnO.

From a technological perspective, the results provide basic guidelines for tailoring solution-processed ZnO for different optoelectronic applications. Alkali-metal replacement is particularly promising for ultraviolet light emitters because of the enhanced near-band-edge emission and the observation of amplified spontaneous emission. Magnesium provides an effective route for bandgap engineering and wavelength tuning, whereas aluminum is suitable for highly conductive transparent electrodes. In contrast, Fe-, Ni-, Cu-, and Pb-doped ZnO may be advantageous where highly compensated or resistive ZnO layers are required.

Overall, this work demonstrates that metal doping provides a versatile and scalable strategy for engineering the electronic functionality of nanocrystalline ZnO. The systematic relationships established between dopant chemistry, carrier concentration, defect population, and optical response provide a general framework for the rational design of ZnO-based materials for future transparent electronics, ultraviolet photonics, sensing technologies, and energy-related applications. Due to current limitations, direct dopant-site analysis, Hall data, defect spectroscopy, and device-level testing were not performed, indicating that further research is needed for a better understanding of ZnO metal doping and its application perspectives.

## Figures and Tables

**Table 1:** Lattice parameters (a and c), c/a ratio, and microstrain (ε) of undoped and metal-ion-doped ZnO thin films, as determined from XRD analysis using the Halder–Wagner method. The values in parentheses represent the uncertainty in the last digit(s).

Sample	Doping (%)	a (Å)	c (Å)	c/a	Microstrain ε (%)
ZnO	0	3.253(4)	5.203(3)	1.599	0.29(4)
Li	1	3.255(2)	5.207(3)	1.598	0.18(6)
	2	3.253(4)	5.212(4)	1.601	0.17(3)
	4	3.254(9)	5.211(7)	1.602	0.18(8)
	8	3.249(3)	5.210(6)	1.601	0.21(3)
Na	1	3.249(8)	5.203(5)	1.601	0.45(3)
	2	3.250(2)	5.204(9)	1.601	0.42(5)
	4	3.251(4)	5.205(7)	1.601	0.67(2)
	8	3.250(8)	5.206(4)	1.602	0.73(4)
K	1	3.253(2)	5.204(7)	1.600	0.28(7)
	2	3.253(8)	5.206(1)	1.600	0.30(3)
	4	3.256(3)	5.206(4)	1.599	0.31(6)
	8	3.257(1)	5.207(2)	1.599	0.21(5)
Mg	1	3.254(5)	5.204(2)	1.599	0.23(7)
	2	3.255(2)	5.204(8)	1.599	0.53(3)
	4	3.256(4)	5.204(6)	1.598	0.38(9)
	8	3.256(8)	5.205(2)	1.598	0.42(3)
Fe	1	3.249(0)	5.206(2)	1.600	0.51(2)
	2	3.254(4)	5.212(6)	1.602	0.48(2)
	4	3.253(2)	5.224(2)	1.606	0.72(6)
	8	3.262(5)	5.238(8)	1.606	0.84(9)
Ni	1	3.258(4)	5.206(5)	1.600	0.49(7)
	2	3.264(3)	5.217(5)	1.602	0.47(5)
	4	3.269(7)	5.216(3)	1.600	0.62(3)
	8	3.281(2)	5.229(1)	1.605	0.87(3)
Cu	1	3.249(7)	5.207(2)	1.602	0.49(6)
	2	3.249(2)	5.208(2)	1.603	0.52(5)
	4	3.251(2)	5.210(3)	1.603	0.78(3)
	8	3.252(8)	5.208(5)	1.601	0.67(8)
Pb	1	3.255(5)	5.217(8)	1.603	0.77(3)
	2	3.257(5)	5.228(8)	1.605	0.89(2)
	4	3.256(3)	5.225(4)	1.605	0.54(2)
	8	3.258(4)	5.231(3)	1.606	1.04(6)
Al	1	3.251(3)	5.206(3)	1.601	0.53(3)
	2	3.248(6)	5.205(3)	1.602	0.47(1)
	4	3.248(2)	5.204(4)	1.602	0.63(6)
	8	3.247(6)	5.204(1)	1.602	0.57(8)

## Data Availability

The original contributions presented in this study are included in the article. Further inquiries can be directed to the corresponding authors.
